# Combination chemotherapy for advanced colorectal cancer

**DOI:** 10.1038/sj.bjc.6600848

**Published:** 2003-04-01

**Authors:** M Mason, P Johnson, R Rudd, the independent members of the MRC Gastrointestinal & Gynaecological Cancer Trials Steering Committee

**Affiliations:** 1 Section of Clinical Oncology, Velindre Hospital, Whitchurch, Cardiff, South Glamorgan CF4 7XL, UK; ^2^CRC Wessex Medical Oncology Unit, Southampton General Hospital, Southhampton, UK; ^3^Department of Medical Oncology, St Bartholomews Hospital, London

**Sir**,

The National Institute for Clinical Excellence (NICE) guidelines for the use of irinotecan, oxaliplatin and raltitrexed in the treatment of patients with advanced colorectal cancer were published in March 2002 (NICE, 2002). In summary, they recommended the use of second-line irinotecan following failure of 5-fluorouracil (5FU) and folinic acid (FA), but withheld their decision on the use of first-line combination chemotherapy until the results of the MRC CR08/FOCUS trial were available.

FOCUS is a five-arm trial assessing whether combination chemotherapy (the modified de Gramont (MdG) schedule of 5FU/FA+irinotecan, or MdG+oxaliplatin) should be given first-line, or second-line following the failure of single-agent MdG. The control arm is first-line MdG followed, at relapse, by single-agent irinotecan, now the NICE approved UK standard.

In June 2002, an editorial in the *British Journal of Cancer* (Saunders and Valle, 2002) and a letter to the Daily Telegraph (Cunningham *et al*, 2002) criticised the NICE recommendations, on the grounds that there was already sufficient evidence to recommend first-line combination chemotherapy. This has implications for the FOCUS study, as two-thirds of patients entering the trial initially receive single-agent MdG. It is disappointing, given the potential damage to an open MRC trial, that the concerns expressed in your editorial had not been made known to any of the groups involved with its conduct. All MRC funded trials are regularly reviewed by an independent Data Monitoring and Ethics Committee (DMEC), who report to a Trial Steering Committee (TSC), which includes an independent chair and members. The DMEC and TSC are independent of the FOCUS management group and NICE. The role of these committees is to ensure that MRC trials remain valid in the face of emerging evidence from other studies, and are not open to the criticisms of being unethical, outdated or irrelevant. The editorial implied, we hope unintentionally, that these responsibilities had not been met in respect of FOCUS.

As independent members of the TSC, we wish to state that FOCUS remains a valid study, and that on the evidence available patients in this trial are not being disadvantaged. This evidence is as follows:

## THE DMEC REPORT

The DMEC for the FOCUS trial met on 14 June 2002, when 1006 of the planned 2100 patients had been entered. In making their recommendations, they considered not only the interim FOCUS data but also all the relevant external evidence. They reported to the TSC that there were no safety or ethical reasons to close or materially change the design of the trial.

To further reassure clinicians and patients, the TSC have agreed to release the information that the current overall median survival for patients in the FOCUS trial is 16 months, which compares favourably with the best arms of most recent randomised trials in this illness (de Gramont *et al*, 2000; Douillard *et al*, 2000; Saltz *et al*, 2000).

## EXTERNAL EVIDENCE

Although two trials comparing first-line 5FU+irinotecan with 5FU alone showed a significant survival benefit with the combination chemotherapy (Douillard *et al*, 2000; Saltz *et al*, 2000), there are difficulties with extrapolating the results of both of these. Firstly, combination chemotherapy causes increased toxicity, and the doses of treatment used in the Saltz trial (Saltz *et al*, 2000) had to be reduced in a subsequent study owing to unacceptable toxicity (Rothenberg *et al*, 2001; Sargent *et al*, 2001). Secondly, the control arms might not be optimal. The Mayo Clinic 5FU/FA schedule was used in one trial (Saltz *et al*, 2000), which is known to be suboptimal (de Gramont *et al*, 1997), and in neither trial was there a specified second-line treatment. It is questionable whether the survival benefits would have been seen, had a control arm been used of the type employed in FOCUS.

Other recent studies are also difficult to extrapolate. Although the Tournigand trial (Tournigand *et al*, 2001) reported impressive median survival, this may have been because of patient selection as a first-line single-agent control arm was not included. Two further trials reported at ASCO this year (Goldberg *et al*, 2002; Grothey *et al*, 2002) do not clarify the situation owing to their design, for example comparing infusional 5FU/FA+oxaliplatin with bolus 5FU/FA+irinotecan.

As NICE has now approved irinotecan as second-line therapy, there is a concern that in FOCUS the crossover for patients will be asymmetric. Patients initially having oxaliplatin could expect to crossover (i.e. receive irinotecan as salvage therapy), whereas those initially on irinotecan would not be able to crossover to oxaliplatin, as it is not licensed or approved in this indication. To address this situation the Trial Management Group (TMG) has suggested that, in those patients fit enough to receive it, a planned crossover treatment be designed. The TSC approved this request at their meeting on 19th June, and this will be implemented as soon as possible. This will ensure that every patient entering FOCUS will potentially have access to irinotecan and oxaliplatin at some point.

The TSC, DMEC and TMG believe that FOCUS remains a key trial despite the studies quoted in your editorial. We support its continued accrual, and encourage existing participants and new centres to enter patients. Only in this way can we clarify the use and sequencing of combination chemotherapy as quickly as possible.
